# FDG-PET/CT-based treatment response predicts recurrence and overall survival in locally advanced cervical cancer

**DOI:** 10.1186/s12880-026-02772-8

**Published:** 2026-09-18

**Authors:** Maria Markus, Hanna Sartor, Maria Bjurberg, David Minarik, Elin Trägårdh

**Affiliations:** 1 Clinical Physiology and Nuclear Medicine, Skåne University Hospital, Lund University, Inga Marie Nilssons gata 47, Malmö, 205 02 Sweden; 2 Wallenberg Centre for Molecular Medicine, Lund University, Lund, Sweden; 3 Diagnostic Radiology, Department of Translational Medicine, Lund University, Skåne University Hospital, Lund, Sweden; 4 Department of Hematology, Oncology and Radiation Physics, Skåne University Hospital, Malmö, Sweden; 5 Department of Clinical Sciences, Lund University, Lund, Sweden; 6 Radiation Physics, Department of Hematology, Oncology and Radiation Physics, Skåne University Hospital, Lund, Sweden

**Keywords:** FDG-PET/CT, Cervical cancer, Treatment response evaluation, EORTC

## Abstract

**Background:**

[18 F]-fluorodeoxyglucose (FDG) positron emission tomography combined with computed tomography (PET-CT) is used for staging and treatment response evaluation in patients with locally advanced cervical cancer. While the maximum standardised uptake value (SUVmax) is a commonly used PET parameter, volume-based parameters (metabolic tumour volume (MTV) and total lesion glycolysis (TLG)) may offer superior predictive value. This study aimed to investigate the predictive value of different treatment response outcomes assessed with PET-CT, using both standard and volume-based PET parameters.

**Methods:**

We retrospectively analyzed 133 patients with locally advanced cervical cancer treated with (chemo)radiotherapy who underwent PET-CT pre- and post-treatment. SUVmax, MTV and TLG were semi-automatically extracted from all scans, including tumour, lymph nodes and metastases. Treatment response was classified as complete metabolic response (CMR) (complete resolution of FDG uptake in all lesions), partial metabolic response (PMR) (> 25% reduction of SUVmax/MTV/TLG), progressive metabolic disease (PMD) (> 25% increase of SUVmax/MTV/TLG or appearance of new FDG-avid lesions) or stable disease (SD) (does not qualify for CMR, PMR or PMD). The three PET-parameters were analysed separately. PMR and SD were analysed as one group due to limited number of events. Associations between treatment response and overall survival (OS) as well as recurrence (binary variable) were analysed through Cox and logistic regression models, respectively.

**Results:**

For recurrence (binary variable), the odds ratio (OR) was 1.2 for patients with PMR/SD for SUVmax, MTV and TLG, while in the group of patients with PMD, it was 1.7 for SUVmax and 1.8 for MTV and TLG, using CMR as reference (i.e. for CMR, the OR is 1.0). The hazard ratio (HR) for OS was 4.0 for patients with PMR/SD and 16.0 for those with PMD, based on SUVmax; for MTV and TLG, HRs were 2.9 for patients with PMR/SD and 16.0 for those with PMD, using CMR as reference (HR 1.0). C-index from adjusted Cox models were comparable: 0.84 for SUVmax, and 0.86 for both MTV and TLG.

**Conclusion:**

FDG-PET/CT-based assessment of treatment response is a strong predictor of recurrence and overall survival in locally advanced cervical cancer. SUVmax, MTV, and TLG demonstrated similar predictive performance.

## Background

Cervical cancer is the fourth most common female malignancy worldwide [1]. [^18^F]fluorodeoxyglucose (FDG) positron emission tomography combined with computed tomography (PET-CT) is commonly used for staging and treatment response evaluation in locally advanced cervical cancer.

With the increasing availability of PET-CT worldwide, the latest revision of the Federation International of Gynaecology and Obstetrics (FIGO) guidelines has included the use of PET-CT to assess pelvic and retroperitoneal lymph node metastases for staging [2]. A follow-up PET post treatment is commonly performed in order to detect remission, therapy failure, early recurrence or unexpected distant metastases ([3–4]).

The most used PET parameter in clinical praxis is currently the maximum standardized uptake value (SUVmax), which measures the hottest voxel in a region of interest (ROI). Volumetric PET parameters such as the metabolic tumour volume (MTV) and total lesion glycolysis (TLG), which take the entire metabolic tumour volume into account, theoretically better represent tumour biology and are therefore also thought to be better diagnostic and prognostic biomarkers [5].

In clinical practice as well as in many studies, treatment response according to PET-CT is often reported through qualitative or visual assessment. Therapy response assessment tools such as the European Organization for Research and Treatment of Cancer (EORTC) criteria have been developed to enable a quantitative and systematic approach on response assessment ([6–7]).

Even though it has previously been shown that PET-CT has diagnostic and prognostic value for evaluating treatment response in patients with locally advanced cervical cancer [8], it must be acknowledged that this group of patients is heterogenous in terms of prognosis, and it remains unclear how PET-CT-protocols and image analysis should best be conducted. A better understanding of this and an optimization of the use of PET-CT could render higher diagnostic and prognostic precision, which is desirable in order to aid clinicians in their choice of treatment of individual patients.

However, it remains unclear whether volume-based PET parameters (MTV and TLG) provide clinically meaningful prognostic information beyond the routinely used SUVmax when treatment response is assessed according to standardized EORTC response criteria.

The aim of this study was to compare the prognostic performance of SUVmax, MTV and TLG for PET/CT-based treatment response assessment using EORTC criteria in patients with locally advanced cervical cancer.

## Methods

### Study type

This is a retrospective, observational cohort study, investigating the predictive value of treatment response assessed with PET-CT [9].

### Patient selection

All patients with cervical cancer who were referred for treatment with (chemo)radiotherapy with curative intention according to national guidelines [10] to the Department of Oncology at Skåne University Hospital, Sweden, were eligible for inclusion. Patients were required to have undergone two PET-CT scans, one prior to treatment and one as follow-up approximately 5 months after treatment. Those who for some reason did not undergo both PET-CT scans (e.g. moved to other address, could not undergo the scanning procedure (e.g. claustrophobia)) were thus not included. No specific exclusion criteria apart from this were stipulated. One patient was excluded during image analysis due to necrosis of the cervix and the adjacent urinary bladder wall, with subsequent contamination of the cervix with FDG-containing urine. Patients were included consecutively between 2011 and 2021, resulting in a total of 133 patients. The study population was the same as in our previous study [11].

### PET-CT imaging

Images were acquired using three different PET-CT systems: Discovery MI (GE Healthcare, Milwaukee, WI, USA), Discovery 690 (GE Healthcare, Milwaukee, WI, USA) or Philips Gemini TF (Philips Healthcare, Cleveland, OH, USA). A standard dose of 4 MBq/kg body weight of [^18^F]FDG was administered intravenously. Accumulation time before imaging was 60 min. Fasting time for at least 4 h and a glucose level of ≤ 180 mg/dl was required before examination.

The baseline-PET was performed from the upper thigh level to the base of the skull, usually complemented by a high-dose CT from the abdomen to upper thigh level for radiotherapy treatment dose planning, anatomic orientation and attenuation correction, combined with a low-dose CT from the base of the skull to the thorax.

The follow-up examination was constituted by a whole-body PET from upper thigh level to the base of the scull, combined with either a low-dose CT or a diagnostic CT with intravenous and oral contrast, depending on patient age and recent previous diagnostic imaging. In general, patients were analysed with the same PET-CT system before and after treatment.

### Image analysis

The HERMES Software System (Hermes Medical Solutions, Stockholm, Sweden) was used to analyse the PET-CT scans retrospectively. Volume segmentation was performed on the primary tumour, local lymph node metastases (defined as pelvic lymph nodes up to the aortic bifurcation), distant lymph node metastases (all other lymph nodes), and distant organ metastases. The sum of all lesions was denoted total tumour burden. Lesions were segmented semi-automatically when possible, using the fixed relative threshold method, defined as 41% of the SUVmax of the lesion. Some cases were not possible to segment semi-automatically and required manual delineation, due to e.g. heterogeneous tumour mass, proximity to the FDG-containing urine bladder, or small lesions with low signal to background ratio. The PET-parameters SUVmax, MTV and TLG (MTV * SUVmean) were obtained from the segmented volumes of interest.

### Metabolic response assessment

For response evaluation, an increase or decrease from the baseline scan was calculated (%). For each patient, treatment response was assessed according to the EORTC criteria [12]. Patients were categorized as complete metabolic response (CMR) (complete resolution of FDG uptake in all lesions), partial metabolic response (PMR) (greater than 25% reduction in SUVmax/MTV/TLG), progressive metabolic disease (PMD) (more than 25% increase in SUVmax/MTV/TLG or appearance of new FDG-avid lesions) or stable disease (SD) (does not qualify for CMR, PMR or PMD). For MTV and TLG, increase or decrease was assessed for the sum of all lesions (tumour, lymph nodes and/or distant metastases). Whenever there was an increase from zero (i.e. no uptake on the first PET-scan), the difference was labelled PMD. Whenever there were new lesions or metastases, the patient was considered PMD regardless of the calculated difference value. PMR and SD were grouped together before analysis (see statistics).

### Statistics

All statistical analyses were performed using R v.4.1.0.

Treatment response was categorized into three different groups: CMR, PMD and PMR/SD. Due to the limited number of events (death/recurrence) in the study population, PMR and SD were grouped together. Kaplan-Meier curves were used to illustrate differences in OS between response groups. Cox regression analysis was used for survival analysis, adjusted for age at first PET-CT and FIGO stage. Harrel’s C-index analysis was performed to evaluate the performance of the survival prediction model. Logistic regression analysis was performed for recurrent disease for the same variables and groups, and was used since the date of the recurrence was not known (only as binary variable). A P-value of < 0.05 was set as threshold for significance for all study outcomes.

## Results

Patient characteristics including age, FIGO stage, histology and follow-up time are shown in Table 1. A total of 37 patients were examined both pre and post- treatment with the GE Discovery 690 system, 37 patients with GE Discovery MI and 44 with Philips Gemini. Fifteen patients performed the first and second scan on different systems.

**Table 1:** Patient characteristics

	Alive	Dead	Total
N	104	29	133
FIGO, n (%)			
IA1	2 (1.9)	0 (0.0)	2 (1.5)
IA2	1 (1.0)	0 (0.0)	1 (0.8)
IB1	10 (9.7)	1 (3.4)	11 (8.3)
IB2	16 (15.5)	7 (24.1)	23 (17.4)
IIA1	7 (6.8)	1 (3.4)	8 (6.1)
IIA2	4 (3.9)	2 (6.9)	6 (4.5)
IIB	47 (45.6)	7 (24.1)	54 (40.9)
IIIA	1 (1.0)	1 (3.4)	2 (1.5)
IIIB	6 (5.8)	6 (20.7)	12 (9.1)
IVA	1 (1.0)	2 (6.9)	3 (2.3)
X	8 (7.8)	2 (6.9)	10 (7.6)
Missing	0	1	1
FIGO, n (%)			
I + II	87 (84.5)	18 (62.1)	105 (79.5)
X + III+IV	16 (15.5)	11 (37.9)	27 (20.5)
Missing	0	1	1
Histology, n (%)			
SCC	79 (76.0)	23 (79.3)	102 (76.7)
AC	25 (24.0)	4 (13.8)	29 (21.8)
ASC	0 (0)	1 (3.4)	1 (0.8)
NEC	0 (0)	1 (3.4)	1 (0.8)
Missing	0	0	0
Recurrent disease, n (%)	11 (10.6)	26 (92.9)	37 (28.0)
Missing	0	1	1
Age	48 [23, 86]	52 [24, 85]	48 [23, 86]
Missing	0	0	0
Follow-up time, years	3.1 [0.4, 8.8]	1.5 [0.7, 3.8]	2.7 [0.4, 8.8]
Missing	0	0	0

Follow-up-time calculated from 1^st^ PET-CT until death, censoring or end-of-follow-up.

Unless otherwise stated data is presented as median [range]. FIGO: The international federation of gynaecology and obstetrics. SCC: Squamous cell carcinoma. AC: Adenocarcinoma. ASC: Adenosquamous carcinoma. NEC: Neuroendocrine carcinoma

Treatment response, categorized as either CMR, PMR/SD or PMD, assessed with each PET-variable is shown in Table 2.

**Table 2:** PET-parameters, data presented as number and percent

*N*	Alive	Dead	Total
	104	29	133
**SUV**_**max**_			
CMR	54 (53.5)	4 (13.8)	58 (44.6)
PMR or SD	35 (34.7)	10 (34.5)	45 (34.6)
PMD	12 (11.9)	15 (51.7)	27 (20.8)
Missing	3*	0	3*
**MTV**			
CMR	54 (53.5)	4 (13.8)	58 (44.6)
PMR or SD	35 (34.7)	7 (24.1)	42 (32.3)
PMD	12 (11.9)	18 (62.1)	30 (23.1)
Missing	3*	0	3*
**TLG**			
CMR	54 (53.5)	4 (13.8)	58 (44.6)
PMR or SD	35 (34.7)	7 (24.1)	42 (32.3)
PMD	12 (11.9)	18 (62.1)	30 (23.1)
Missing	3*	0	3*

* Patients with no uptake on either first or second PET

TLG: total lesion glycolysis. MTV: metabolic tumour volume. SUVmax: maximum standardized uptake value. CMR: complete metabolic regression. PMR: partial metabolic regression. SD: stable disease. PMD: progressive metabolic disease

**Fig. 1:**
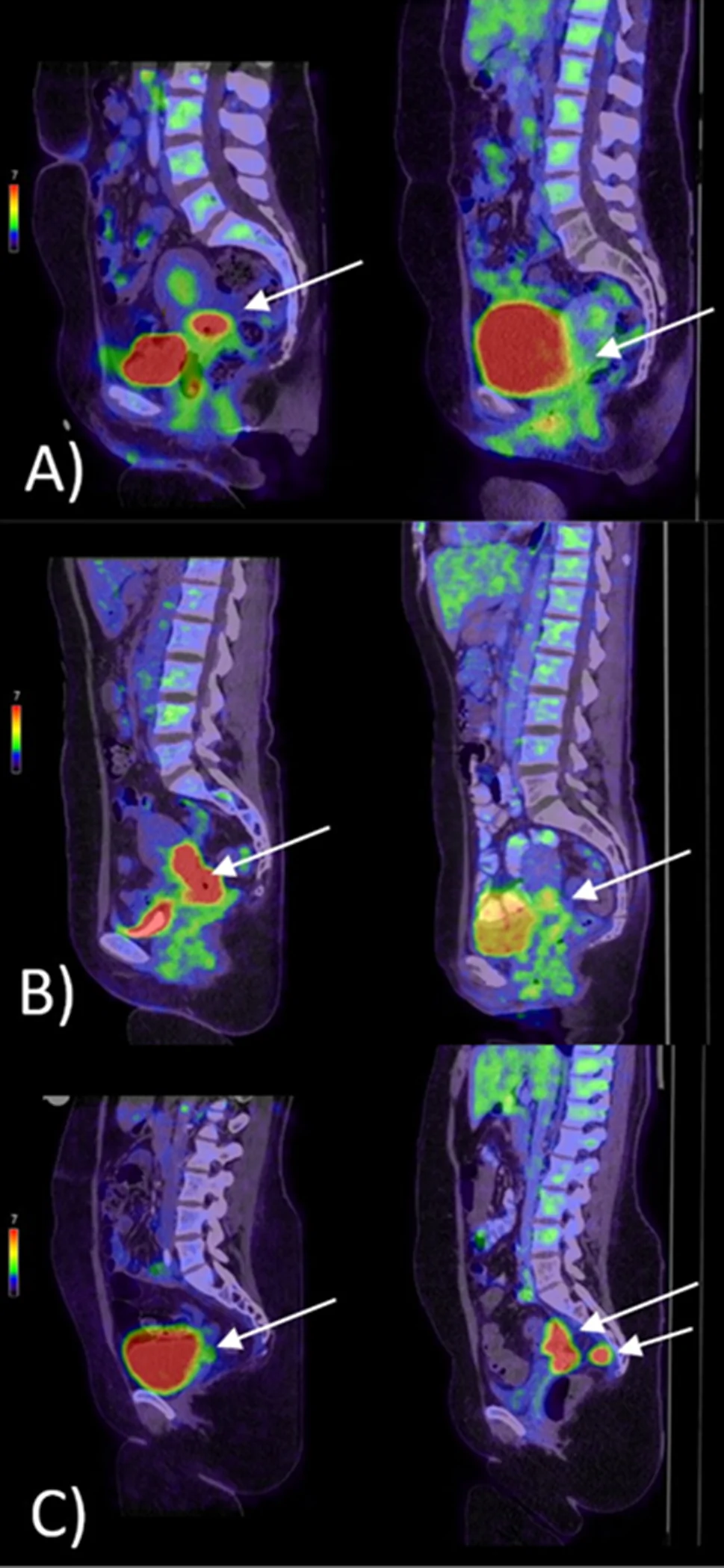
Fused PET-CT images of patient with (**A**) CMR (**B**) PMR and (**C**) PMD (as measured with any of the PET-parameters). Pre-treatment to the left and post-treatment to the right. White arrow indicates tumour (and metastasis in case C)) localisation. CMR: complete metabolic response, PMR: partial metabolic regression, PMD: progressive metabolic disease

Examples of patients with three different treatment outcomes (CMR, PMR and PMD) are shown in Fig. 1A-C, respectively.

### Recurrence

Analyses comparing groups of treatment response in relation to recurrent disease showed little difference between the PET-parameters (adjusted values) (Table 3).

**Table 3:** Unadjusted and adjusted* logistic regression for recurrent disease, evaluating each variable one at a time

Variable	OR (95% CI)	*p*-value	OR_Adj*_ (95% CI)	*p*-value
**SUV**_**max**_		< 0.001		< 0.001
CMR	1.0 (Reference)		1.0 (Reference)	
PMR or SD	1.2 (1.1–1.4)		1.2 (1.1–1.5)	
PMD	1.7 (1.4–2.1)		1.7 (1.4–2.1)	
**MTV**		< 0.001		< 0.001
CMR	1.0 (Reference)		1.0 (Reference)	
PMR or SD	1.2 (1.0–1.4)		1.2 (1.0–1.4)	
PMD	1.8 (1.5–2.1)		1.8 (1.5–2.1)	
**TLG**		< 0.001		< 0.001
CMR	1.0 (Reference)		1.0 (Reference)	
PMR or SD	1.2 (1.0–1.4)		1.2 (1.0–1.4)	
PMD	1.8 (1.5–2.1)		1.8 (1.5–2.1)	

*Adjusted for age and FIGO X+III+IV vs I+II. TLG: total lesion glycolysis. MTV: metabolic tumour volume. SUVmax: maximum standardized uptake value. CMR: complete metabolic regression. PMR: partial metabolic regression. SD: stable disease. PMD: progressive metabolic disease

CMR was used as reference, so that odds ratio (OR) for recurrent disease for the CMR group was 1.0. The OR for recurrent disease in the PMR/SD group was 1.2 (95% CI 1.1.-1.5) for SUVmax and 1.2 (95% CI 1.0-1.4) for MTV and TLG. The OR for the PMD group was 1.7 (95% CI 1.4–2.1) for SUVmax and 1.8 (95% CI 1.5–2.1) for both TLG and MTV. Logistic regression analysis for recurrence depending on treatment response (unadjusted and adjusted for age and FIGO stage) showed a statistically significant association (*p* < 0.001) for all three PET-parameters.

### Survival

Cox regression analysis (unadjusted and adjusted for age and FIGO stage) for mortality depending on treatment response is shown in Table 4.

**Table 4:** Unadjusted and adjusted* Cox regression for mortality, evaluating each variable one at a time

Variable	HR (95% CI)	*p*-value	HR_Adj*_ (95% CI)	*p*-value
**SUV**_**max**_		< 0.001		< 0.001
CMR	1.0 (Reference)		1.0 (Reference)	
PMR or SD	3.3 (1.0–11)		4.0 (1.2–13)	
PMD	16 (5.3–49)		16 (5–49)	
**MTV**		< 0.001		< 0.001
CMR	1.0 (Reference)		1.0 (Reference)	
PMR or SD	2.4 (0.7–8.2)		2.9 (0.8–10)	
PMD	17 (5.8–52)		16 (5.3–48)	
**TLG**		< 0.001		< 0.001
CMR	1.0 (Reference)		1.0 (Reference)	
PMR or SD	2.4 (0.7–8.2)		2.9 (0.8–10)	
PMD	17 (5.8–52)		16 (5.3–48)	

*Adjusted for age and FIGO X+III+IV vs I+II

Follow-up-time calculated from 1^st^ PET-CT until death, censoring or end-of-follow-up. TLG: total lesion glycolysis. MTV: metabolic tumour volume. SUVmax: maximum standardized uptake value. CMR: complete metabolic regression. PMR: partial metabolic regression. SD: stable disease. PMD: progressive metabolic disease

Patients with PMD had higher hazard ratio (HR) for mortality than patients with PMR/SD, independent of PET-parameter used. CMR was used as reference (HR for CMR for mortality = 1.0). The results were similar between PET-parameters (adjusted values): the HR for the PMR/SD group was 4.0 (95% CI 1.2–13) for SUVmax and 2.9 (95% CI 0.8–10) for both TLG and MTV. The HR for the PMD group was 16 (95% CI 5–49) for SUVmax and 16 (95% CI 5.3–48) for both TLG and MTV. There was a statistically significant association (*p* < 0.001) between treatment response and mortality for all three PET-parameters.

**Fig. 2:**
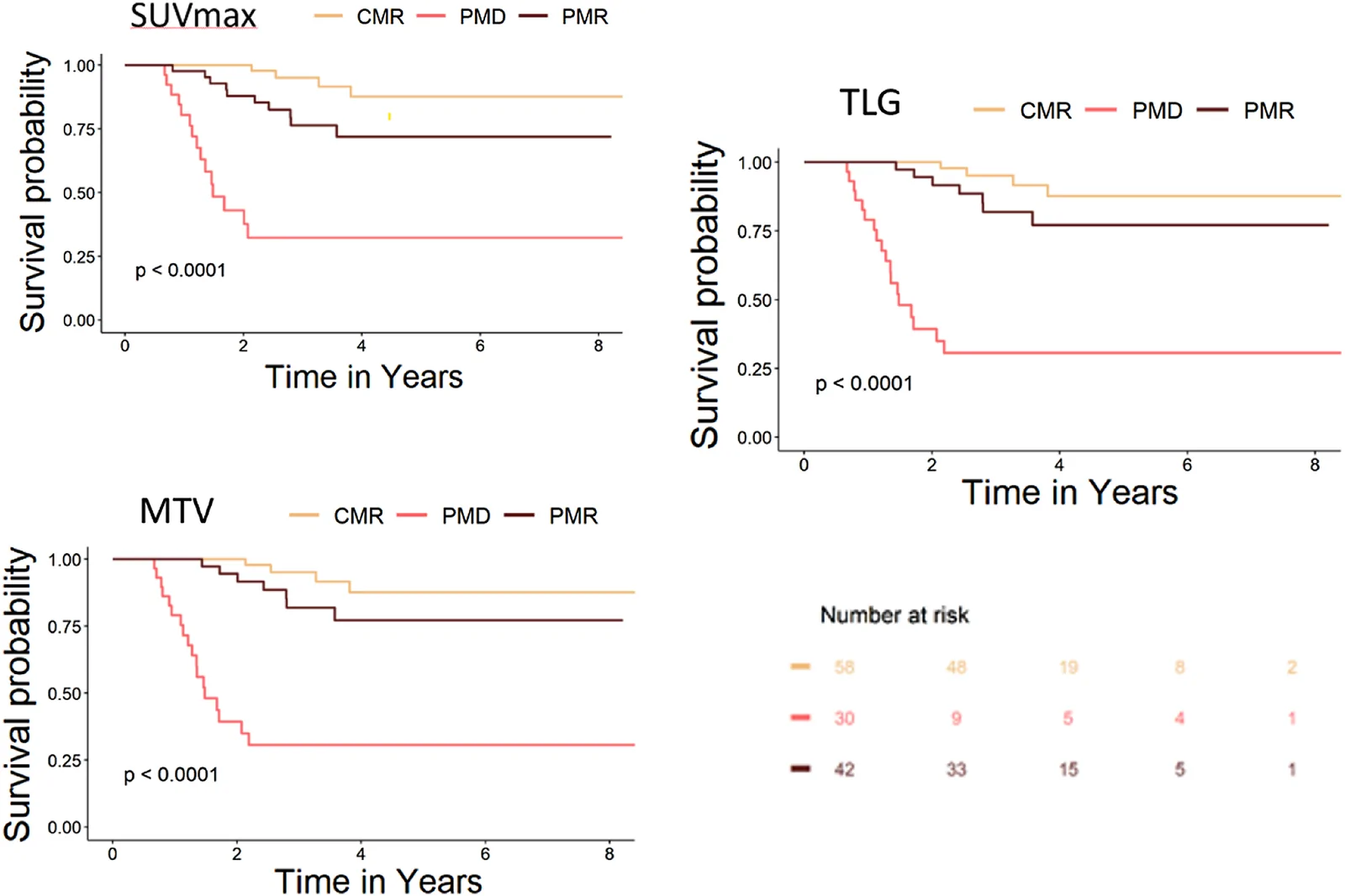
Kaplan-Meier curves demonstrating overall survival in relation to treatment response assessed with PET-CT, for each response group (CMR, PMR or SD, or PMD. Survival curves are for treatment response assessed with **A**) SUVmax, **B**) MTV and **C**) TLG. SUVmax: maximum standardized uptake value SUVmax. MTV: metabolic tumour volume. TLG: total lesion glycolysis. CMR: complete metabolic response. PMR: partial metabolic regression, SD: stable disease PMD: progressive metabolic disease

Kaplan-Meier survival curves for the different treatment response groups as assessed with the different PET-parameters are shown in Fig. 2, indicating only slight differences between PET-parameters. Likewise, C-index values for prediction of mortality were similar for the three different PET-parameters (Table 5).

**Table 5:** Harrell’s concordance index (C-index) from adjusted Cox models

SUV_max_	0.838
MTV	0.857
TLG	0.857

TLG: total lesion glycolysis. MTV: metabolic tumour volume. SUV_max_: maximum standardized uptake value

## Discussion

This study demonstrates that treatment response assessment using PET-CT is a significant predictor of both recurrence and OS in women with locally advanced cervical cancer. As anticipated, patients with PMD exhibited the highest risk of recurrence and mortality. Notably, our results revealed minimal differences in predictive performance among the three PET parameters SUVmax, MTV, and TLG. For instance, OR for recurrence in the PMD group was 1.7 for SUVmax and 1.8 for both MTV and TLG, with overlapping confidence intervals. Similarly, HR for mortality in the PMD group was 16 across all PET parameters. Kaplan-Meier survival curves and Harrell’s C-index values further supported the comparable predictive utility of these parameters.

Our findings reinforce the predictive value of metabolic imaging in this patient population, as they are in line with previous reports [13–18]. However, due to methodological heterogeneity, our results are not directly comparable with other studies investigating recurrence or OS. Several earlier groups dichotomized treatment response outcome assessed with PET-CT, for example into CMR versus non-CMR, due to small sample sizes. For instance, Lima et al. reported significant OS differences between these two groups in a cohort of 82 patients, using the EORTC criteria [15]. One study including 36 patients with recurrent cervical cancer showed no difference in OS between patients with PMD versus those without progression [19]. In a prospective study by Schwarz et al. [20] including 92 women, three response groups were compared, as in our study. They found a much higher HR for mortality in the PMD group compared to our study (HR = 32.6). In their study, treatment response was assessed visually and had a shorter time interval between examinations. Another study by Scarsbrook et al. used a qualitative 5-point scoring system (equivalent to the Deauville score system used for treatment response assessment in lymphoma). They also found treatment response to be predictive of survival, with a visual divergence in Kaplan-Meier curves between the five response groups, indicating heterogeneity in the group of patients with no-CMR [21].

Although the predictive potential of PET-CT has already been ascertained, the reproducibility of the findings is important, counteracting publication bias as well as contributing to the much-needed growing body of evidence [22].Importantly, the results of this study give a deeper understanding of the properties of different PET-parameters and the optimal use of PET-CT in this heterogenous population of patients.

Although SUVmax remains the most used parameter due to its simplicity, observer independence and ease of application, volume-based metrics such as MTV and TLG may provide additional biological insights ([23–24]). Despite their theoretical advantages, our results did not demonstrate any predictive benefits of MTV or TLG over SUVmax, which may support continued use of SUVmax for routine clinical treatment response assessment, without requiring more time-consuming volume-based measurements. We speculate that the lack of superiority might be due to an inherent covariation between the PET-parameters and tumour properties where a high glucose metabolism reflected by a high SUVmax is more informative than tumour volume. Also, the EORTC criteria used is a categorical scale with four categories and moreover, only three groups were compared in this study. Patients who were classified into the same treatment response group may still have had a large variation in the percentual change in SUVmax, MTV and TLG respectively on pre vs. post treatment PET-CT scans, so that the true difference between the PET parameters may be larger than indicated by our results.

Appropriate treatment monitoring is essential for the next step in patient management. It is well known that complete response after primary treatment is a positive predictive factor ([20–21, 25–26]), but the group of patients who do not show complete response is heterogeneous. Local inflammation induced by (chemo)radiotherapy can make it difficult to determine whether any remaining FDG uptake is malignant or reactive. As illustrated in our study, there is a divergence in the survival curves of the different treatment response groups. This has clinical implications, as it may indicate the need for different and individualized treatment and follow-up plans for patients who do not demonstrate complete metabolic response.

These findings suggest that PET/CT response assessment may support risk stratification after chemoradiotherapy and help guide the intensity of follow-up and consideration of further treatment, although prospective studies are needed before changes in clinical management can be recommended.

Strengths in our study include the large number of patients included in our study, which allowed us to compare CMR with two other groups, i.e., PMD and PMR/SD – compared to previous studies only being able to compare two groups. Also, we used the quantitative EORTC criteria for treatment response assessment, which have been recommended in order to avoid inter- and intra-reviewer variability and to facilitate the reproducibility of studies [7, 12].

A few methodological issues require consideration. First, the size of the study population forced us to stratify into three instead of the four different response groups. Larger study cohorts are required in order to enable stratification into the four response groups defined by the EORTC criteria. Second, in our study population, different PET systems with different reconstruction algorithms were used. This may have introduced inter-patient variability when analysing the PET parameters in those cases where the patient had their pre- and post-treatment examinations performed on different PET/CT scanner. However, only 15 out of 133 had their pre- and post treatment exam performed on different systems. Furthermore, the use of different PET systems makes our cohort more representative in a real world setting where different PET-CT systems are used. Third, we acknowledge that there was no comparison with methods such as CT or MRI, which is still the standard work-up in many sites internationally. This was however not the scope of interest for this study. Fourth, tumour, lymph node, and metastasis segmentations were done mainly semi-automatically, but in some cases manual segmentation was necessary. Manual segmentations are observer-dependent which limits the reproducibility of the procedure [24]. Fifth, in our register, we lacked data on time-to-event for recurrence. This forced us to use logistic instead of Cox regression analysis. Lastly, when analysing the post-treatment FDG-PET/CT, there may have been mixed responses or clinical progression within each tumour/lymph node/metastatic site that were not taken into consideration, as only the pooled PET parameter values are shown.

## Conclusion

FDG-PET/CT-based assessment of treatment response is a strong predictor of both recurrence and overall survival in patients with locally advanced cervical cancer. Our findings demonstrate that the predictive value is consistent across SUVmax, MTV, and TLG, suggesting that more complex and time-consuming volume-based parameters may not offer additional clinical benefit over the simpler SUVmax.

## Data Availability

The datasets used in this study are available from the corresponding author on reasonable request.

## Abbreviations

FDG: [18 F]-Fluorodeoxyglucose
PET: Positron emission tomography
CT: Computed tomography
FIGO: Federation international of gynaecology and obstetrics
SUV: Standardized uptake value
MTV: Metabolic tumour volume
TLG: Total lesion glycolysis
EORTC: European organization for research and treatment of cancer
CMR: Complete metabolic response
PMR: Partial metabolic response
PMD: Progressive metabolic disease
